# Vaccine Safety Surveillance Systems: Critical Elements and Lessons Learned in the Development of the US Vaccine Safety Datalink’s Rapid Cycle Analysis Capabilities

**DOI:** 10.3390/pharmaceutics5010168

**Published:** 2013-03-12

**Authors:** Robert L. Davis

**Affiliations:** Director of Research, Center for Health Research Southeast, 3495 Piedmont Rd NE, Suite 110, Atlanta, GA 303035, USA; E-Mail: Robert.l.davis@kp.org; Tel.: 404-364-7197

**Keywords:** vaccine safety, drug safety, surveillance

## Abstract

Since the late 1990s, there have been tremendous strides made in improving the capacity for carrying out routine active surveillance of new vaccines in the United States. These strides have led to new surveillance systems that are now in place. Some of the critical elements that are part of successful vaccine or drug safety surveillance systems include their use of (i) longitudinal data from a discrete enumerated population base, (ii) frequent, routine transfers of small amounts of data that are easy to collect and collate, (iii) avoidance of mission creep, (iv) statistical capabilities, (v) creation of an “industrialized process” approach and (vi) political safe harbor.

## 1. Background

The Vaccine Safety Datalink (VSD) project was started in 1990 as a collaborative research enterprise between the Centers for Disease Control and Prevention (CDC) and four health maintenance organizations (HMOs) for the purpose of enabling studies into serious adverse events following immunization [1]. The number of participating HMOs grew first to eight and then 10 HMOs, with a covered population of over nine million members enrolled at any given time (and over 24 million individuals cumulatively during the project’s lifespan) [2]. 

In the VSD, patient encounters and diagnoses can be linked to previous vaccine or drug exposures via a patient-specific study ID. This enables patient level data to be merged across many different administrative files, including, for example, enrollment files, which track when subjects are or are not enrolled in the HMO, an outcomes file, which tracks what diagnoses are made for each subject in each HMO (along with when and where the diagnosis was made), and a procedures file, which tracks surgeries and other procedures. Data on medications and vaccinations received are also tracked in separate files and can be linked on the individual patient level to enrollment and encounter data, thereby creating a dataset that can be used to assess the temporal relationship between medication or vaccine exposure and new onset (or exacerbation) of specific conditions or diseases. 

The VSD is an active safety surveillance system and was constructed in large part to address the limitations of passive surveillance systems, such as the Vaccine Adverse Events Reporting System (VAERS). As a passive surveillance system, VAERS receives reports of events, which are voluntarily submitted by those who experience them or by their caregivers or from others [3]. The limitations of these passive surveillance systems include underreporting of vaccine adverse events, reporting of temporal associations or unconfirmed diagnoses, lack of denominator data and, finally, the lack of unbiased comparison groups. As a result, understanding causal relationships between vaccines and adverse events solely from VAERS reports is usually not possible.

While systems for vaccine safety surveillance exist in other countries and regions—notably the United Kingdom, Canada, Scandinavia and parts of Asia—the Vaccine Safety Datalink is one of the largest ongoing networks that is population-based and expressly focused on vaccine safety surveillance [4,5,6,7,8]. The unbiased ascertainment of outcomes and ready availability of appropriate comparison groups makes the VSD particularly useful for conducting post-licensure vaccine safety studies, and it corrects for the methodological weaknesses found in passive systems, such as VAERS [3].

Once established, the size of the VSD network enabled CDC and HMO researchers to undertake a series of seminal research projects that evaluated the risk of new (and existing) vaccines, and it also allowed researchers the ability to quickly respond to new concerns about vaccine safety. Such studies conducted by the VSD included ones that outlined the risk of seizures following whole-cell pertussis or measles-mumps-rubella vaccine, found a lack of association between thimerosal containing vaccines and risk for poor neurodevelopment or for autism and analyzed the risk for idiopathic thrombocytopenic purpura ITP after measles, mumps and rubella (MMR) vaccination [9,10,11,12].

Around the time these studies were underway, a number of high-profile public health controversies occurred relatively close in time. Because these controversies centered around prescription medications and vaccinations, the Food and Drug Administration (FDA), the CDC and others began exploring ways of accruing data closer to “real-time” in order to speed the recognition of potential safety problems. One such event was the withdrawal of rofecoxib (Vioxx) from the U.S. market in 2004, even though (as some have argued) unacceptable cardiovascular risks were observable up to four years earlier [13,14]. Because of the delay in identifying and acting upon the increased risk, thousands of patients continued to be prescribed rofecoxib and placed at increased risk of myocardial infarction or sudden cardiac death. Another significant public health challenge occurring around the same time was the licensure and recommendation for the tetravalent rhesus-human reassortant rotavirus (Rotashield) vaccine to be administered to all U.S. infants at 2, 4 and 6 months of age [15,16]. Once available in late 1998, the uptake of Rotashield was brisk, but by July 1999, reports of intussusception to the Vaccine Adverse Events Reporting System (VAERS) suggested a potential serious problem. Formal epidemiologic studies found that the risk of intussusception 3–14 days following vaccination was over 20-fold higher than among non-vaccinated infants [17,18]. Between the time of licensure of Rotashield and its voluntary withdrawal from the U.S. market, over 500,000 infants had received at least one dose of vaccine. 

As a result of both these issues, there was considerable interest from federal agencies in developing the capacity for detecting vaccine or medication adverse events closer to “real-time” [19]. In addition, it was recognized that, once enabled and if large enough, an ongoing monitoring system that did not detect problematic signals could be useful for reassuring the public and regulatory agencies about the relative safety of new vaccines or medicines. Work by investigators with the CDC and the VSD in 2003 developed such a “proof-of-concept” system that appeared to perform satisfactorily. To first build and test this new system, the first step was to use historical VSD data and reconstruct what “would have been seen” by a monitoring system had the data been available weekly from each of the HMOs. These new “rapid cycle analysis” programs (as they were quickly named) were able to relatively easily identify the reassortant rotavirus vaccine (RVV)-intussusception association and was able also to rapidly identify the relative safety of newly introduced acellular pertussis vaccines compared to the old whole-cell vaccine [20].

In 2005, the VSD formally integrated the Rapid Cycle Analysis (RCA) process into its ongoing funded work, with a goal to monitor adverse events following vaccination in near real-time. RCA was used to monitor newly licensed vaccines and to monitor the effects of new vaccine recommendations. The choice of adverse events to monitor was based on pre-licensure study data, any early signals from the Vaccine Adverse Event Reporting System or concerns raised in published scientific studies [21].

Since its introduction into the VSD, RCA has been used to monitor a number of vaccines and has had a number of significant successes, two of which are related here. The first example centers on the use of RCA in monitoring the newly licensed quadrivalent meningococcal conjugate vaccine (Menactra). Menactra was licensed in 2005 and was recommended by the CDC and the Advisory Committee on Immunization Practices (ACIP) for use among adolescents 11–12 years old [22]. Uptake of the vaccine was brisk, but shortly after its introduction, a small, but worrisome, number of cases of Guillain-Barré Syndrome (GBS) following vaccination were reported to the Vaccine Adverse Events Reporting system (VAERS) [23]. This cluster of case reports for such an unusual condition immediately raised the concern of whether these cases were related to vaccination (as opposed to other factors, such as a circulating virus). To help address these questions, additional information was available from the VSD, since RCA surveillance activities for Menactra had been instituted with the release of the vaccine. No signal of an increased risk for GBS following vaccination was seen in the VSD’s RCA program, and while the numbers of vaccinated persons were still relatively small, the quick availability of the data (and the lack of a signal) was reassuring. Over the next few weeks, reports from VSD’s RCA system continued to show no increase in the rate of GBS among vaccines [24]. These reports, along with other supplementary data, suggested that a considerably larger study would be necessary for a more definitive assessment of Menactra’s safety profile. Importantly, though, the data from the VSD RCA activities provided timely reassurance to federal agencies, with the ultimate result being that vaccination activities were able to continue nationally.

A second demonstration of RCA’s active surveillance benefit was seen during surveillance of the new combination measles, mumps, rubella and varicella vaccine (MMRV, ProQuad, Merck & Co., Inc.), licensed in 2005. As part of the normal RCA surveillance activities that were planned for MMRV, an increase in risk for seizures (almost all febrile seizures) was observed and peaked during the time window of 7–10 days after vaccination. The fact that an increase in seizure risk was observed following vaccination was not unexpected—after all, the VSD had already found this in previous VSD studies of MMR back in 2001 [9]. What was unexpected, though, was the observation that children vaccinated with MMRV were at double the risk for seizures compared with children vaccinated with MMR and varicella vaccine delivered separately (RR 1.98; 95% CI 1.43, 2.73) [25]. These findings were validated by confirmatory analyses, the ACIP changed their recommendations regarding the use of MMRV and the CDC recommended that MMR vaccine and varicella vaccine be administered separately for the first dose in the age group of 12–47 months (unless the parent or caregiver expressed a preference for the combination MMRV vaccine). 

## 2. Critical Elements of a Successful Surveillance System

The following sections should be read with the understanding that they represent my subjective interpretations of otherwise objective events. With that in mind, these are my thoughts with regards to a number of the critical elements, which have contributed to the successful functioning of this new active vaccine safety surveillance system.

### 2.1. Longitudinal Data from Discrete Population Bases, Which Have Been Specially Curated for Research Use

Given the relative rarity of many of the adverse events, which are routinely studied by the VSD, it may seem self-evident that there is a fundamental need by these systems to have access to data from very large populations. Beyond this, however, there are some additional requirements so that routine and repeated epidemiologic evaluations can occur and provide timely and relevant information for policy makers. For instance, the data used for these surveillance activities needs to be readily available longitudinally, such that groups or cohorts of individual vaccines can be followed up over time. This ensures that the temporal relationship between exposure (in this case, vaccine) and outcome (for example, seizures) can be assessed and, when necessary, evaluated with some degree of granularity (for example, seizures in the 7–10 day time window following MMRV vaccination). Comprehensive collection of outcome data is also desirable, so that, for example, if a subject receives a vaccination in the doctor’s office and subsequently seeks care in the emergency department for a possible adverse reaction (*i.e.*, swollen limb, high fever, seizures, *etc.*), the outcomes are identified regardless of where medical care is sought. In the case of the CDC’s RCA system, outcome information is available from diagnoses made in an outpatient clinic, emergency department and hospital. In addition, at some sites, supplemental data on diagnoses can be gleaned by additional data files, such as laboratory values, radiology results, procedures, referrals, *etc*.; visits out of plan are captured by claims data. Since vaccine or drug safety surveillance systems fundamentally rely upon complete follow-up of patients after vaccination or drug exposure for their validity, data streams, which capture data from each of these systems, is optimal. 

As noted above, the VSD links patient encounters and diagnoses with previous vaccine or drug exposures via patient-specific study IDs, thereby creating datasets able to study temporal relationships between medication or vaccine exposure and new onset (or exacerbation) of specific conditions or diseases. It is important to recognize that these files contain a considerable amount of confidential health information about HMO members and, so, its protection is of utmost importance. For this reason, the data are maintained behind firewalls and are accessed only by specific research employees of each institution. Data that can be used to identify patients do not traverse the firewall to different institutions; instead, a central data coordinating center typically distributes programming code to each site, and then, each site extracts and transfers the minimal amount of study-specific data back to the lead site for aggregation and analysis.

The files listed above (*i.e.*, encounter, enrollment, vaccine, drug and procedure files) are just a few of many research data files that are created by extracting data from routinely collected administrative data files at each HMO. These source administrative files do not inherently contain “research quality” data, and considerable quality assessment work is required before the administrative data is ready to be used for research. It is not unusual for VSD research and surveillance activities to require a number of data programmers to work full-time on distilling and producing research quality data from otherwise routinely collected HMO administrative data. 

In terms of limitations, if medications or immunizations are disbursed or administered outside of the HMO and not submitted for reimbursement, the records of these occurrences may be missing from the electronic medical files. Finally, temporal associations between vaccinations and events that occurred sooner than 24 h after vaccination (such as anaphylaxis) cannot always be distinguished with certainty. This is because, in many cases, if the exposure (vaccination) and outcome (anaphylaxis) are both recorded on the same day in the electronic files, it is not always possible (without medical record review) to delineate clearly whether the exposure preceded the event.

### 2.2. Simple Data

Surveillance systems often perform most efficiently when they rely on easily collected, limited amounts of data that are simple to produce and easy to transfer. Just as important is to ensure that the data remain de-identified, thereby eliminating the possibility of accidentally disclosing protected patient healthcare information. When the VSD RCA surveillance program was in its early stages and first being developed, these ideas were somewhat contentious and their implementation took a bit of getting used to. A large reason for this is that, before the RCA project became a routine aspect of the VSD’s workload, investigators, programmers and analysts were more accustomed to working on and analyzing very sophisticated datasets that had been produced for hypothesis testing. Such datasets may have taken considerable time to construct and might have needed multiple iterations before the final analyses were performed. By contrast, the rapid cycle surveillance activities depended upon the production of weekly datasets at each site, with the data itself being limited to only a very few (*i.e.*, 5–10) data variables, such as number of individuals vaccinated during a given week and the number or “count” of vaccinated individuals with specific outcomes, with each of these counts being stratified by age and gender. 

### 2.3. Mission Creep

A common feature of good researchers is that they are rarely satisfied with the data on hand and frequently want to ask additional questions or get more data to explore alternative hypotheses in order to have a better idea of what is happening. When faced with a vaccine adverse event “signal”, for example, an investigator may have a series of additional questions and may decide he/she would like to look at the data broken down by gender, by ethnicity, by different age groups or by various types of comorbidities (*i.e.*, diabetes, hypertension) *etc.* This is a form of “mission creep” and, if not addressed head-on, may eventually morph the entire nature of the surveillance activities into something more akin to a hypothesis-generating or hypothesis-testing apparatus. Once this happens, the time and programmer full-time equivalent (FTE) needs required from each HMO to create more expansive (and no longer so simple) weekly datasets will likely increase, the costs related to these rapid-cycle activities will increase and the fundamental surveillance activities may eventually be relegated to the background.

In order to achieve compliance with requests for frequent transfers of data, we found that it was important to resist the temptation to expand the scope of the routine data extracts and to limit the data to only the minimal amount necessary for clearly delimited surveillance activities.

### 2.4. Statistical Expertise

During the first days of developing active surveillance efforts, the scientific efforts were concentrated primarily on deciding upon the most appropriate methodology that could accommodate the type of data provided and that could account for the repeated (weekly) testing of the data for “signals”. We elected to use sequential probability ratio tests (SPRT), an analytic approach that has been used by industry to monitor process performance and is typically used for situations where continuous monitoring is desirable [26]. SPRT’s performance characteristics suggested that it was more sensitive than other available statistical processes.

To aid our methods development process, we chose the rotavirus vaccine and the whole cell pertussis vaccines for proof-of-concept work, reasoning that a reasonably good surveillance system should have the capability of detecting the level of adverse event risks associated with these vaccines. To our consternation, however, we quickly found that the whole cell diphtheria, tetanus, and pertussis (DTP) vaccine appeared to have a lesser risk for (presumed febrile) seizures among children compared with the acellular DTP vaccines. (This was contradictory to what was expected, based on pre-licensure vaccine studies of the acellular pertussis vaccine). After examining the crude data from each site, it became apparent that strong secular trends in seizure rates (most likely due to increased data collection capabilities instituted by the health plans) were strong enough to completely reverse the lower risk for seizures with acellular DTP vaccine compared with whole cell vaccine. It was only after we carefully controlled for these time trends that we were able to observe the lower risk for seizures attendant with the changeover to DTaP [20].

A more perplexing challenge arose when we began surveillance on a new combination childhood vaccine introduced at one of the HMOs. We evaluated this new vaccine for both a 20% increase and a two-fold increase in risk (relative risks of 1.2 and 2.0, respectively), as we had little *a priori* evidence to guide our choice of risk level. Surprisingly, we found that the system signaled for a two-fold increase in risk, but at the same time, when evaluating for a 20% increase in risk, the system signaled that the vaccine was not associated with an increased risk above 1.0. This made little sense at first: how could a vaccine be associated with a doubling of risk at the same time that it was indicating no increase in risk? This led to the uncomfortable situation where investigators might come to opposite conclusions regarding the safety of a particular vaccine, solely depending on which level of relative risk they had chosen *a priori* to evaluate [27]!

Upon closer examination of both the data at hand and the theory behind the SPRT methodology, an explanation was found. Consider a situation where the true (but unknown) risk following vaccination was slightly (20%) elevated (*i.e.*, true relative risk (RR) was 1.2). Under this scenario, had we evaluated the data using a surveillance limit of 1.2, we would have found that there was more evidence supporting the alternative relative risk of 1.2 compared to the null hypothesis of no increased risk (RR of 1.0). However, had we evaluated the data using a surveillance limit of 2.0, we would have found the opposite—specifically, we would have found that there was more evidence supporting the null hypothesis of no increased risk (RR of 1.0) than for the alternative RR of 2.0. In the first scenario, the surveillance system would have signaled a safety problem, but in the second scenario, the surveillance system would have signaled no concern. The only difference, again, was the strength of the signal we chose to look for. 

Since it was not tenable to have the accuracy and sensitivity of the surveillance system depend on our *a priori* choice of risk level, one of the biostatisticians, Martin Kulldorff, developed a methodology that instead estimated the true elevation in risk observed from the data itself. This methodology, called maxSPRT, uses a composite alternative hypothesis, with the relative risk defined simply as being greater than one (rather than taking on a specific value). The maxSPRT methodology is quite straightforward, requiring only that the user specify alpha and an upper limit on the length of surveillance. Use of the maxSPRT methodology resolves the problems outlined above and is now the standard employed by the VSD active surveillance system and has been used for weekly surveillance of meningococcal, tetanus-diphtheria-pertussis, rotavirus, measles-mumps-rubella-varicella, human papillomavirus and influenza vaccines (both seasonal and H1N1) [27,28,29,30]. 

The take-home message from this experience (and others not outlined here) was that having advanced expertise in biostatistical methodology was critical to the ongoing success of the VSD surveillance activities. Rather than the surveillance activities being “plug and play” (as sometimes assumed by researchers and others outside the network), the challenges faced in combining, understanding and correctly studying the complicated, sophisticated data coming from 10 disparate HMOs scattered across the United States required substantial time and effort from a number of biostatisticians.

### 2.5. Consistent Funding and Resource Stewardship

There is an implicit—but often overlooked—challenge for surveillance activities. Namely, funding announcements and opportunities in the United States (from NIH, *etc.*) are episodic by nature and are typically oriented to stimulate scientific discovery rather than for the purposes of safety surveillance. In addition, surveillance activities essentially need to take on an “industrial process” mindset, whereby vaccine-specific surveillance activities can continue, even in the midst of other unrelated public health crises or other vaccine safety concerns that might preoccupy agency leadership and divert resources. By nesting these surveillance activities within the CDC’s Vaccine Safety Datalink project—which was funded on 10 year cycles—the scientists within the active surveillance team of the VSD were able to concentrate on science rather than on the pursuit of funding. As a result, the CDC’s RCA was able to employ monitoring activities as new vaccines were planned for licensure or to develop novel methodologies when needed. 

Separate from the CDC’s ability to consistently fund and maintain the RCA activities, a significant concern surrounding the VSD surveillance activities (and around any such surveillance activity), was the possibility that the “noise”, that is, the number of false signals, from the system would overwhelm response capabilities and that time, resources and energy would be needlessly expended on following up on initial signals of false concern, resulting in less resources being available for evaluating real safety concerns. Because of these legitimate concerns, the VSD surveillance team examined the signal performance of the RCA system and recently published data on the results of investigating ten signals, which occurred over three years of active surveillance. Yih *et al*. showed that one signal ultimately led to a change in national vaccine policy, while nine other signals were investigated and found to be due to non-safety concerns [31]. Similar to the example discussed earlier concerning DTP, most of the nine other signals were dismissed fairly quickly as due to data errors, confounding, changes in coding over time or other reasons. (A more complete discussion of these signals is presented in the excellent article by Yih *et al.*).

### 2.6. Political Safe Harbor

Finally, by its very nature, the CDC vaccine safety surveillance system is geared to identify possible safety problems related to vaccines, which are brought to market only after a considerable investment of time, energy and money. It is necessary for investigators to be able to independently investigate—without outside influence—the signals leading to safety concerns. Investigators also need the freedom and independence to discuss their findings openly in scientific forums, without pressure to arrive at particular conclusions that might have substantial economic, policy or political ramifications. To its credit, the CDC’s active surveillance activities have been able to successfully navigate the fine line between being responsive to additional queries into its findings from many different sources, while maintaining the independence necessary for presenting its findings in the public forum and to publish in high impact journals. 

## 3. Conclusions

In summary, the experiences of the VSD active surveillance activities have led to the development of new statistical methodologies, and its findings have had a positive influence on public health. Critical system components contributing to its success included a commitment to the use of simple data, the ability to create, transfer and use relatively small amounts of data on a routine schedule (e.g., weekly, monthly or quarterly) and use of standardized data fields across HMOs. In addition, the VSD has had critical access to comprehensive population-based vaccination and healthcare utilization/outcome data at the individual person-level. Finally, the ongoing contribution of a number of investigators with advanced biostatistical expertise, along with stable funding that enabled multiple rounds of developmental activities—was critical to the development and success of the VSD’s surveillance activities. This overall model has been adopted by the new FDA Sentinel Initiative project, which has been tasked with overseeing drug safety surveillance activities for a combined population of over 130 million persons in the United States. 
